# EFFECTS OF TOPICAL TREATMENT WITH EUPHORBIA TIRUCALLI LATEX ON THE SURVIVAL AND INTESTINAL ADHESIONS IN RATS WITH EXPERIMENTAL PERITONITIS

**DOI:** 10.1590/S0102-6720201500040006

**Published:** 2015

**Authors:** Lilhian Alves de ARAÚJO, Fátima MRUÉ, Roberpaulo Anacleto NEVES, Maxley Martins ALVES, Nelson Jorge da SILVA-JÚNIOR, Marcelo Seixo de Brito SILVA, Paulo Roberto de MELO-REIS

**Affiliations:** 1 Postgraduate in Master Degree in Environmental and Health Sciences, Pro-Rectory of Postgraduate Studies and Research, Area V, Campus I, the Catholic University of Goiás; 2 Department of Veterinary Medicine, Veterinary School, Campus Samambaia, Federal University of Goiás, Goiânia, GO, Brazil

**Keywords:** Peritonitis, Euphorbia tirucalli, Survival

## Abstract

***Background*:**

The use of plants of the family Euphorbiaceae, particularly *Euphorbia tirucalli* (avelós) has been popularly widespread for treating a variety of diseases of infectious, tumoral, and inflammatory.

***Aim*:**

To demonstrated antimicrobial and immunomodulatory effects of these extracts, evaluating the effect of a topical treatment with an aqueous solution of avelós latex on the survival and on intestinal adhesions in rats with experimental peritonitis.

***Methods*:**

Peritonitis was induced in 24 Wistar rats, that were randomized into four groups of six as follows: (1) Control group (n=6), no treatment; (2) Antibiotic group (n=6), treatment with a single intramuscular dose of antibiotic Unasyn; (3) Saline group (n=6), the abdominal cavity was washed with 0.9% saline; and (4) *E.tirucalli* group (n=6), the abdominal cavity was washed with *E. tirucalli* at a concentration of 12 mg/ml. The animals that died were necropsied, and the time of death was recorded. The survivors were killed on postoperative day 11, and necropsy was subsequently performed for evaluation of the intestinal adhesions.

***Results*:**

Significant differences were observed in the control and antibiotic groups (p<0.01) with respect to the survival hours when compared with the saline and *E. tirucalli* groups. There was no significant difference (p>0.05) in the survival of animals in the saline and*E. tirucalli* groups; however, one animal died in the saline group. Necropsy of the animals in the saline and *E. tirucalli*groups showed strong adhesions resistant to manipulation, between the intestinal loops and abdominal wall. The remaining groups did not show any adhesions.

***Conclusions*:**

Topical treatment with *E. tirucalli* latex stimulated an increased formation of intestinal adhesions and prevented the death of all animals with peritonitis.

## Abstract (pt)

**Racional:**

O uso de plantas da família Euphorbiaceae, principalmente a *Euphorbia tirucalli* (avelós), tem sido popularmente difundido para o tratamento de uma variedade de doenças de natureza infecciosa, tumoral e inflamatória.

**Objetivo:**

Avaliar o efeito do tratamento tópico com a solução aquosa do látex do avelós na sobrevida e nas aderências intestinas de ratos com peritonite experimental.

**Métodos:**

Foi induzido peritonite em 24 ratos Wistar e randomizados em quatro grupos de seis, assim distribuídos: 1) Controle - (n=6), nenhum tratamento; 2) Antibiótico - (n=6), tratamento com dose única intramuscular de antibiótico Unasyn (Pfizer - São Paulo); 3) Salina - (n=6), lavagem da cavidade abdominal com solução fisiológica 0,9%; 4) *E.Tirucalli *- (n=6), lavagem da cavidade abdominal com *E. tirucalli* na concentração de 12 mg/ml. Os animais que morreram foram submetidos à necropsia e o horário do óbito anotado. Os sobreviventes foram submetidos à eutanásia no 11^o^dia de pós-operatório e, posteriormente, realizou-se a necropsia para avaliação da formação de aderências.

**Resultados:**

Os grupos controle e antibiótico obtiveram diferença significativa (p<0,01) com relação às horas de vida entre os grupos salina e *E. tirucalli*. Não houve diferença significativa (p>0,05) na sobrevida dos animais dos grupos salina e *E. tirucalli, *no entanto, houve um óbito no grupo salina*.* A necropsia dos animais dos grupos salina e *E. tirucalli* mostrou aderências firmes e resistentes à manipulação entre alças intestinais e parede abdominal. Os demais grupos não tiveram formação de aderências.

**Conclusão:**

O tratamento tópico com o látex da *E. tirucalli* estimulou maior formação de aderências intestinais e evitou o óbito de todos animais com peritonite até o período avaliado.

## INTRODUCTION

Peritonitis is a serious disease, due to the inflammatory response in the serous membrane lining the abdominal cavity and viscera. The immediate answers to peritonitis are hyperthermia, bowel distension, hyperemia, accumulation of gases and liquids, hypovolemia and pain. At the same time, there are cardiac, respiratory, renal and metabolic responses. It is also high contribution of fibroblasts that produce fibrin, responsible for the formation of intra-abdominal adhesions^17^^,^ 20 ^,^ 21 ^,^ 28.

Although often the treatment of peritonitis include mechanical removal of contaminants through peritoneal washings with saline, antibiotics and abdominal integrity restoration associated with modern intensive and surgical care units, currently peritonitis still accounts for approximately 50% of deaths consequent to sepsis^1^^,^ 7 ^,^ 12 ^,^ 19.

The *Euphorbia tirucalli*, the Euphorbiaceae family, is a plant used in folk medicine. From Africa was brought to Brazil with ornamental purposes, it is commonly known as avel*ó*s. It produces white colored latex widely used by Brazilian folk medicine to treat injuries, infectious diseases, tumors and inflammatory diseases^2^^,^ 9 ^,^ 25 ^,^ 29 ^,^ 30.

In scientific research, the latex has shown immunomodulatory activity^3^^,^ 5 ^,^ 13 and also the ethanol extract of *E. tirucalli* used in vitro in various concentrations, showed antimicrobial activity against various bacteria strains, among them *Escherichia coli*, which has fundamental importance for being one of the most frequent bacterial species found in fecal peritonitis^10^^,^ 11 ^,^ 16 ^,^ 23 ^,^ 26.

Given the popular use of *E. tirucalli* in treating diseases and previous research demonstrating antimicrobial and immunomodulatory effects, this study aimed to evaluate the effect of topical treatment with the aqueous solution of avel*ó*s latex in survival and in the intestinal adhesions in mice with fecal experimental peritonitis.

## METHODS

### Animals used for testing

For the study, 24 rats (*Rattus norvegicus*) Wistar male adult were used, with body weight ranging from 200 to 300 g, coming from the Central Animal Laboratory at the Catholic University of Goiás and aged between two and three months. The experiment was approved by the institution's Ethics Committee, protocol 006/2012 and followed according to international standards and Brazilian Society of Science in Animal laboratory - SBCAL.

### Botanical certification of Euphorbia tirucalli

The plant *E. tirucalli* was at the Experimental Studies Laboratory of Biotechnology of the Postgraduate Master degree program in Environmental Sciences and Health at the Catholic University of Goiás (LEB / MCAS-PUC Goiás) (-16 ° 40 '32.79 "- 49 ° 14' 38.58 "). The botanical identification of the sample used in the experiment was performed by Dr. Joseph Angelo Rizzo, the Institute of Biological Sciences, Federal University of Goiás (ICB-UFG). A voucher specimen was deposited in the herbarium of the institution, with the registration number 47797.

### Latex of Euphorbia tirucalli Dilution

The sap was extracted through an incision in the trunk and branches of the adult plant, and then collected using disposable syringe, weighed and immediately transferred to a sterile glass beaker containing distilled water. The initial concentration was 0.1 ml corresponding to 120 mg pure latex. After dilution in 9.9 ml of distilled water, the final concentration was 12 mg/ml. This final concentration was established during toxicological evaluation in a previous experiment. The quality of the solution was determined by absence of clots and their homogeneity. This material was stored at 4° C for a maximum of 30 days^14^^,^ 15.

### Induction of peritonitis

The animals were anesthetized in the anterior muscle of the right thigh, with ketamine hydrochloride 10% (Syntec - Veterinary Service) at a dose of 12.5 mg/kg of animal weight. Subsequently, it was injected into the upper left quadrant of the abdomen solution of 5 ml/kg of fresh faeces of the animals (2 g) diluted in 17 ml saline. Before injection, the suspension was filtered through gauze in order to allow the passage of the inner needle toward the cavity^6^.

### Blood collection and laboratory analysis

To confirm the diagnosis of peritonitis, 0.5 ml of blood was collected from the caudal vein of mice with insulin heparinized syringe after disinfection with 70% alcohol and transferred to tube with ethylenediamine tetraacetic acid. After, total leukocyte count were performed (Neubauer chamber, New Optics, São Paulo, Brazil) and differential in smears stained with panotic, viewed in light microscope (Nikon, Eclipse Model E-200).

### Experimental procedure

Six hours after induction of peritonitis with stool suspension injection, 24 mice were randomized into four groups: 1) Control (n=6), no treatment; 2) Antibiotic (n=6), treatment with a single intramuscular dose of antibiotic Unasyn (Pfizer, England) 30 mg; 3) Saline (n=6), the abdominal cavity washed with saline solution 0.9%; 4) *E.tirucalli* (n=6), the abdominal cavity washing with *E. tirucalli* latex at a concentration of 12 mg/ml.

In Saline and *E. tirucalli* groups rats were anesthetized intramuscularly in the anterior aspect of the right thigh, with mixture of Xylazine 2% (Syntec, São Paulo, Brazil - veterinary use) at a dose of 2.5 mg/kg and hydrochloride ketamine 10% (Syntec, São Paulo, Brazil - veterinary use) at a dose of 50 mg/kg, then underwent laparotomy with about 2 cm long. Subsequently, the solutions used for washing were placed in the abdominal (0.9% saline of the animals in group 3, *E. tirucalli* 12 mg/ml in group 4 in amount of 5 ml and left for 2 min, after this procedure the peritoneal fluid was aspirated and wiped. The abdominal wall was sutured in two planes with mononylon 4-0 and simple running suture.

Animals that died underwent necropsy and at the time of death was noted. The survivors were euthanized with a lethal dose of ketamine on the 11^th^day after surgery and were examined in the abdominal cavity adhesions and possible outbreaks of macroscopic infection. Adhesions were classified into six grades: Grade 0 - no adhesions; Grade 1 - few adhesions, of fibrinous character, easily undone by manipulation; Grade 2 - firm adhesions, resistant to manipulation, between intestinal loops, but not involving the abdominal wall; Grade 3 - strong adhesion, resistant to handling, between the abdominal wall and an organ or structure; Grade 4 - strong adhesion, resistant to handling, between the abdominal wall and over an organ or structure; Grade 5 - firm adhesions, resistant to manipulation, between intestinal loops and abdominal wall with enteric fistula^8^.

### Statistical analysis

In order to compare the differences among the data was used descriptive statistics, ANOVA (analysis of variance) followed by Tukey test, which demonstrates that the difference among groups was significant. For all analyzes it was adopted a significance level of p<0.05. Statistical program Bioestat 5:0^4^ was used.

## RESULTS

### Laboratory analysis

Total and differential leukocyte counts was performed only for the infectious process (peritonitis) prior to the respective treatments. It was demonstrated leukocytosis in all experimental groups compared to hematological reference values (Table 1).

**TABLE 1:** - Descriptive statistics of laboratory analysis among the control group (1), antibiotic (2), salt (3) and E. tirucalli (4)

**Parameters**	**Groups**			
Lymphocytes (103 µL)	1 (Control)	2 (Antibiotic)	3 (Saline)	4 (E.tirucalli)
Mean (±SD)	7579±2034	7848±2556	7131±1305	6568±1145
Variation	3960-9768	5490-11660	5796-9048	5301-8640
Monocytes				
Mean (±SD)	264±121	149±79	301±176	363±45
Variation	112-396	0-260	134-580	282-405
Eosinophils				
Mean (±SD)	0±0	0±0	0±0	0±0
Variation	0-0	0-0	0-0	0-0
Neutrophils Seg.				
Mean (±SD)	3017±568	6202±3039	5166±2979	4342±1500
Variation	2375-3968	3510-10120	1683-8670	2632-6700
Neutrophils bast.				
Mean (±SD)	223±163	0±0	212±181	177±198
Variation	0-396	0-0	0-510	0-405
Basophils				
Mean (±SD)	0±0	16±39	0±0	0±0
Variation	0-0	0-96	0-0	0-0
Total leucocytes				
Mean (±SD)	11100±2402	14233±5599	12783±2533	11233±1633
Variation	6600-13200	9000-22000	9900-17000	9300-13500

SD= standard deviation

### Survival

**FIGURE 1:**
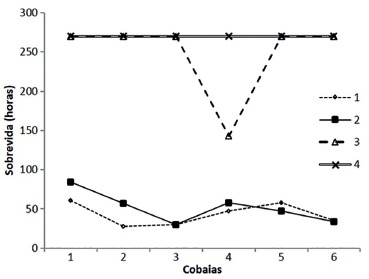
- Survival in hours of Control (1), Antibiotic (2), Saline (3) and *E. tirucalli* (4) groups

The treatments Control and Antibiotic not survived until the 11^th^evaluation day of the experiment (270 h), and the difference between them and other groups, in hours of life, was statistically significant (p<0.01). The groups of Saline and *E. tirucalli* were those who survived to the end of the experiment, and a animal of Saline group died before that time, with 143 hours of life. There was no significant difference (p>0.05) in survival of the other animals of Saline and *E. tirucalli* groups (Figure 1).

### Necropsies

**FIGURE 2:**
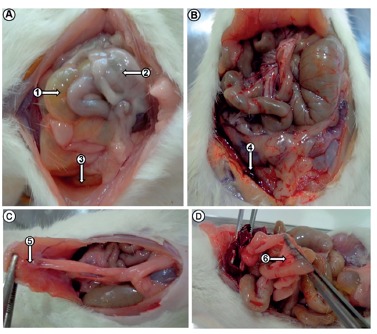
- Autopsy of the animals of the Control (A), Antibiotic (B) Saline (C) and *E. tirucalli* (D) groups: The images demonstrate enterocolonic dilatation (A-1), fibrin (A-2), peritoneal fluid turbid (A-3), hemorrhagic spots in the cavity (B-4), abdominal wall compliance (C-5) and adhesion between the bowel (D-6)

The necropsy of all animals in Control and Antibiotic groups revealed diffuse peritonitis foul-smelling, generalized redness, cloudy peritoneal fluid, fibrin, abscesses and some animals had enterocolonic swelling, necrosis of the liver segments and hemorrhage (Figure 2A-B). The autopsy of the animals in Saline and E. tirucalli groups showed only adhesions between the bowel and abdominal wall, in greater numbers in *E. tirucalli* group (Figure 2C-D). There was no abscess formation in any of the groups.

The degrees of adhesions found in all animals can be seen in Table 2. In the group treated with saline solution was found formation of adhesions Grade 2, and the ones treated with the latex had increased formation of adhesions Grade 3.

**TABLE 2:** - Classification of the degrees of peritoneal adhesions among the six individuals in the Control (1) Antibiotic (2) Saline (3) and*E. tirucalli* (4) groups

Grade*	**Groups/Individuals**	**Total**			
	**Control**	**Antibiotic**	**Saline**	**E.tirucalli**	
0	6	5	3	0	14
1	0	1	2	3	6
2	0	0	1	0	1
3	0	0	0	3	3
4	0	0	0	0	0
5	0	0	0	0	0
Total	6	6	6	6	24

* Grade 0=no adhesions; Grade 1=few adhesions, of fibrinous character, easily undone by manipulation; Grade 2=dense adhesions, resistant to manipulation, between intestinal loops, but not involving the abdominal wall; Grade 3=strong, resistant to manipulation adhesions between abdominal wall and an organ or structure; Grade 4=firm, resistant to manipulation adhesions between abdominal wall and over an organ or structure; Grade 5=dense adhesions, resistant to manipulation, between loops and abdominal wall with enteric fistula^8^.

## DISCUSSION

Plants are rich source of bioactive compounds that can interact with our bodies contributing to the discovery of new drugs and helping therapeutic practices to prevent, cure or subtract the symptoms of diseases^22^.

In this study, the washing of the abdominal cavity with the latex *of E. tirucalli* 12 mg/ml increased the survival of animals with fecal peritonitis to the evaluation period. Similar results were observed in other studies where the death of animals with fecal peritonitis was averted after peritoneal washing with lidocaine^6^ and clorexidine^7^.

It should be noted that in the group that did the peritoneal cavity washing with saline solution (Saline) showed higher survival than the groups Control and Antibiotic. The beneficence on survival of rats with fecal peritonitis, after treatment with peritoneal washing saline solution had already been confirmed and described priorly^24^. It is known that it is used by many surgeons; however, there is still controversies^27^.

The peritoneal washing with saline solution increased the survival of animals as well as washing with *E. tirucalli*; however, with latex there was no death within the study period. Another factor that may have contributed to the increased survival of the animals in Saline and *E. tirucalli* groups was the appropriate time for the start of treatment after induction of peritonitis, consequently shorter infection. The literature mentions best predictors when the therapeutic procedure is initiated from 5 min to 6 h following induction of peritonitis^24^.

During the autopsy of animals for evaluation of adhesions and macroscopic foci of infection were found between Control and Antibiotic groups, diffuse peritoneal signs of infection without adhesions. The animals treated only with intramuscular antibiotic showed no improvement in the clinical outcome, and survival time and necropsy evaluation very similar to the Control group. The choice of antibiotic used in this group was due to its bactericidal activity and proven efficacy against microorganisms likely present in the gastrointestinal tract, commonly indicated for the treatment of secondary peritonitis. However, only systemic single dose antibiotic without direct action on the abdominal contamination was not enough to increase the survival of animals. A similar result was found in the survival of mice with peritonitis treated only with a single dose of intramuscular antibiotics, gentamicin and clindamicin^7^.

In saline and *E. Tirucalli* groups, there was no macroscopic signs of infection, adhesions only, being more firm and resistant to manipulation involving abdominal wall and bowel in *E. tirucalli* group. This may have been another contributing factor in the increased survival of the animals treated with the latex, since adhesion is attributed to the function of isolating septic processes (abscess) and protect the body from bacterial dissemination. Inhibition of these adhesions is accompanied by increased mortality resulting from intra-abdominal septic generalized process^18^.

Additional in vivo studies using models of peritonitis associated with treatment with active ingredients isolated from *E. tirucalli* latex, would be relevant to compare results and detail their beneficial effects in secondary peritonitis.

## CONCLUSION

Treatment with *E. tirucalli* and saline solution washing led the animals to survive the same period, with no deaths in the group treated with the latex in the evaluation period. There was also increased formation of firm intestinal adhesions, resistant to the handling, in the group of animals treated with *E. tirucalli.*
